# Covering functionals of cones and double cones

**DOI:** 10.1186/s13660-018-1785-9

**Published:** 2018-07-24

**Authors:** Senlin Wu, Ke Xu

**Affiliations:** Department of Applied Mathematics, Harbin University of Science and Technology, Harbin, China

**Keywords:** 52A20, 52A15, 52A40, 52C17, Banach–Mazur distance, Compact convex cones, Convex body, Covering, Hadwiger’s covering conjecture

## Abstract

The least positive number *γ* such that a convex body *K* can be covered by *m* translates of *γK* is called the covering functional of *K* (with respect to *m*), and it is denoted by $\Gamma_{m}(K)$. Estimating covering functionals of convex bodies is an important part of Chuanming Zong’s quantitative program for attacking Hadwiger’s covering conjecture. Estimations of covering functionals of cones and double cones, which are best possible for certain pairs of *m* and *K*, are presented.

## Introduction

A compact convex set $K\subseteq\mathbb{R}^{n}$ having interior points is called a *convex body*. The *interior* and *boundary* of *K* are denoted by int*K* and bd*K*, respectively. We write $\mathcal{K}^{n}$ for the set of convex bodies in $\mathbb{R}^{n}$. Concerning the least number $c(K)$ of translates of int*K* needed to cover a convex body *K*, there is a long-standing conjecture:

### Conjecture 1

(Hadwiger’s covering conjecture)

*For each* $K\in\mathcal{K}^{n}$, $c(K)$ *is bounded from above by* $2^{n}$, *and this upper bound is attained only by parallelotopes*.

We refer to [1–4], and [5] for more information and references about this conjecture. Note that, for each $K\in\mathcal{K}^{n}$, $c(K)$ equals the least number of smaller homothetic copies of *K* needed to cover *K* (see, e.g., [1, p. 262, Theorem 34.3]). Therefore, $c(K)\leq m$ for some $m\in \mathbb{Z}^{+}$ if and only if $\Gamma_{m}(K)<1$, where $\Gamma_{m}(K)$ is defined by

$$ \Gamma_{m}(K)=\min\Biggl\{ \gamma> 0: \exists \{{x_{i}}: {i=1,\ldots,m} \}\subseteq\mathbb {R}^{n} \text{ s.t. } K\subseteq\bigcup _{i=1}^{m}(x_{i}+\gamma K)\Biggr\} $$

and called *the covering functional of* *K* *with respect to* *m*. A closely related concept is studied in functional analysis. Given a bounded subset *M* of a normed space *E* with unit ball *B*, the *m-th entropy number* $\varepsilon_{m}(M)$ of *M* is defined by (cf. [6, p. 6–7])

$$ \varepsilon_{m}(M)=\inf \Biggl\{ {\varepsilon>0}: {M\subseteq\bigcup _{i=1}^{m}(\varepsilon B+x_{i})\text{ holds for suitable }x_{1},\ldots,x_{m}\in E} \Biggr\} . $$

When *K* is a translate of the unit ball *B* of a finite-dimensional normed space *E*, we have $\Gamma_{m}(K)=\varepsilon_{m}(K)$.

Clearly, for each $m\in\mathbb{Z}^{+}$, $\Gamma_{m}(\cdot)$ is affinely invariant. More precisely,

$$ \Gamma_{m}(K)=\Gamma_{m}\bigl(T(K)\bigr),\quad \forall T\in \mathcal{A}^{n}, $$

where $\mathcal{A}^{n}$ is the set of non-degenerate affine transformations from $\mathbb{R}^{n}$ to $\mathbb{R}^{n}$. Thus we identify convex bodies that are affinely equivalent, and when writing $\mathcal{K}^{n}$ we are actually referring to the quotient space of $\mathcal{K}^{n}$ with respect to affine equivalence.

For each pair $K_{1}$, $K_{2}$ of convex bodies in $\mathcal{K}^{n}$, the *Banach–Mazur distance* $d_{\mathrm{BM}}(K_{1},K_{2})$ (also called the *Asplund metric*, cf. [7]) between them is defined by

$$ d_{\mathrm{BM}}(K_{1},K_{2}):=\ln\min\bigl\{ \gamma\geq1: K_{1}\subseteq T(K_{2})\subseteq \gamma K_{1}+x, x\in\mathbb{R}^{n}, T\in\mathcal{A}^{n}\bigr\} . $$

Then $(\mathcal{K}^{n},d_{\mathrm{BM}})$ is a compact metric space (cf. [8] and [7]). Zong (cf. [9]) proved that $\Gamma _{m}(\cdot)$ is uniformly continuous on $\mathcal{K}^{n}$. Bezdek and Khan improved this result by showing that $\Gamma_{m}(\cdot)$ is Lipschitz continuous on $\mathcal{K}^{n}$ with $(n^{2}-1)/(2\ln n)$ as a Lipschitz constant (cf. [10]). These results show that each $K\in\mathcal{K}^{n}$ can be covered by at most $2^{n}$ smaller homothetic copies of *K* if and only if

$$ c(n):=\sup \bigl\{ {\Gamma_{2^{n}}(K)}: {K\in\mathcal{K}^{n}} \bigr\} < 1. $$

Due to these facts, estimating covering functionals of convex bodies is an important part of Zong’s quantitative program for attacking Hadwiger’s covering conjecture (cf. [9] for more details).

Our starting point is Theorem 1 in [9]: $\Gamma_{8}(C)\leq \frac{2}{3}$ when *C* is a three-dimensional convex cone (the convex hull of the union of a planar convex body *K* and a singleton not contained in the plane containing *K*). By applying new ideas, we show that this estimation can be improved when $\Gamma_{7}(K)<\frac{1}{2}$, and that this estimation can be extended to higher dimensional situations. Moreover, we also obtain an estimation of $\Gamma_{m}(C)$ when *C* is a double cone.

## Covering functionals of convex cones

We start with an elementary lemma.

### Lemma 1

*Let* $K\subset\mathbb{R}^{n}$ *be a convex set*, $x\in\mathbb{R}^{n}$, *and* $\lambda,\gamma\in [{0},{1} ]$. *Then*

$$ (x+\gamma K)\cap K\subseteq\lambda x+(\lambda\gamma+1-\lambda)K. $$

### Proof

Let *z* be an arbitrary point in $(x+\gamma K)\cap K$. Then $z-x\in \gamma K$ and $z\in K$. Therefore

$$\begin{aligned} z-\lambda x&=\lambda(z-x)+(1-\lambda)z \\ &\in\lambda\gamma K+(1-\lambda)K \\ &=(\lambda\gamma+1-\lambda)K. \end{aligned}$$

It follows that $z\in\lambda x+(\lambda\gamma+1-\lambda)K$. □

For each $\bar{x}\in\mathbb{R}^{n}$, we put $x=(\bar{x},0)\in \mathbb{R}^{n+1}$. Each point $x\in\mathbb{R}^{n+1}$ can be written in the form $(\bar{x},\alpha )$, where $\bar{x}\in\mathbb{R}^{n}$ and $\alpha\in\mathbb{R}$. Let *K* be a convex body in $\mathcal{K}^{n}$ containing the *origin* *o* in its interior, and *p* be a point in $\mathbb{R}^{n+1}\setminus\mathbb{R}^{n}\times\{0\}$. Put $C=\operatorname{conv}((K\times\{0\})\cup\{p\})$, i.e., *C* is the *cone* having *p* as a vertex and whose *base* is *K*.

### Lemma 2

*Suppose that* $m\in\mathbb{Z}^{+}$, $\gamma,\lambda\in(0,1)$, $\mu\in [{\lambda},{1} ]$, $z=\mu(\bar{y},0)+(1-\mu)p$ *for some* $\bar{y}\in K$, *and that* $\{{\bar{u}_{i}}: {i\in[m]} \} \subseteq\mathbb{R}^{n}$ *is a set of points satisfying*

$$ K\subseteq\bigcup _{i\in[m]}(\bar{u}_{i}+\gamma K). $$

*Then there exists* $i\in[m]$ *such that*

$$ z\in\lambda(\bar{u}_{i},0)+(\lambda\gamma+1-\lambda)C. $$

### Proof

We have

1 $$\begin{aligned} z&=\mu(\bar{y},0)+(1-\mu)p=\mu(\bar{y},0)+\frac{1-\mu}{1-\lambda }(1-\lambda)p \\ &=\frac{1-\mu}{1-\lambda}\bigl(\lambda(\bar{y},0)+(1-\lambda)p\bigr)+ \frac {\mu-\lambda}{1-\lambda}(\bar{y},0). \end{aligned}$$

Without loss of generality we may assume that $\bar{y}\in\bar {u}_{1}+\gamma K$. It follows that

2 $$\begin{aligned} & \lambda(\bar{y},0)+(1-\lambda)p \\ &\quad \in \lambda(\bar{u}_{1},0)+\lambda\gamma\bigl(K\times\{0\}\bigr)+(1- \lambda )p \\ &\quad = \lambda(\bar{u}_{1},0)+(\lambda\gamma+1-\lambda) \biggl( \frac{\lambda \gamma}{\lambda\gamma+1-\lambda}\bigl(K\times\{0\}\bigr)+\frac{1-\lambda }{\lambda\gamma+1-\lambda}p\biggr) \\ &\quad \subseteq \lambda(\bar{u}_{1},0)+(\lambda\gamma+1-\lambda )C. \end{aligned}$$

By Lemma 1, we have

$$ \bar{y}\in(\bar{u}_{1}+\gamma K)\cap K\subseteq\lambda\bar {u}_{1}+(\lambda\gamma+1-\lambda)K, $$

which implies that

3 $$ (\bar{y},0)\in\lambda(\bar{u}_{1},0)+(\lambda\gamma+1- \lambda ) \bigl(K\times\{0\}\bigr)\subseteq\lambda(\bar{u}_{1},0)+( \lambda\gamma +1-\lambda)C. $$

From (1), (2), and (3) it follows that $z\in\lambda(\bar{u}_{1},0)+(\lambda\gamma+1-\lambda)C$. □

For two numbers satisfying $0\leq\lambda_{1}\leq\lambda_{2}\leq1$, we put

$$ C_{\lambda_{1},\lambda_{2}}= \bigl\{ {\lambda(\bar{x},0)+(1-\lambda)p}: {\bar{x}\in K, \lambda\in[\lambda_{1},\lambda_{2}]} \bigr\} . $$

It is not difficult to verify that $C=C_{0,1}$. And when $0\leq\lambda_{1}\leq\lambda_{2}\leq1$ and $\lambda_{2}\neq0$, we have

4 $$\begin{aligned} C_{\lambda_{1},\lambda_{2}}&= \bigl\{ {\lambda(\bar{x},0)+(1-\lambda )p}: {\bar{x}\in K, \lambda\in[\lambda_{1},\lambda_{2}]} \bigr\} \\ &=\lambda_{2} \biggl\{ {\frac{\lambda}{\lambda_{2}}(\bar{x},0)+ \frac {1-\lambda}{\lambda_{2}}p}: {\bar{x}\in K,\lambda\in[\lambda _{1}, \lambda_{2}]} \biggr\} \\ &=\lambda_{2} \biggl\{ {\frac{\lambda}{\lambda_{2}}(\bar{x},0)+\biggl(1- \frac {\lambda}{\lambda_{2}}\biggr)p+\biggl(\frac{1}{\lambda_{2}}-1\biggr)p}: {\bar{x}\in K, \lambda\in[\lambda_{1},\lambda_{2}]} \biggr\} \\ &=\lambda_{2} \biggl\{ {\frac{\lambda}{\lambda_{2}}(\bar{x},0)+\biggl(1- \frac {\lambda}{\lambda_{2}}\biggr)p}: {\bar{x}\in K,\lambda\in[\lambda _{1}, \lambda_{2}]} \biggr\} +(1-\lambda_{2})p \\ &=\lambda_{2} \biggl\{ {\lambda(\bar{x},0)+(1-\lambda)p}: {\bar{x}\in K,\lambda\in\biggl[\frac{\lambda_{1}}{\lambda_{2}},1\biggr]} \biggr\} +(1-\lambda _{2})p \\ &=\lambda_{2}C_{\lambda_{1}/\lambda_{2},1}+(1-\lambda_{2})p. \end{aligned}$$

### Lemma 3

*Let* *m* *be a positive integer satisfying* $\gamma=\Gamma_{m}(K)<1$, *and* $0<\lambda_{1}\leq\lambda_{2}\leq1$. *Then* $C_{\lambda_{1},\lambda_{2}}$ *can be covered by* *m* *translates of* $(\lambda_{1}\gamma+\lambda_{2}-\lambda_{1})C$.

### Proof

If $\lambda_{1}=\lambda_{2}$, then $C_{\lambda_{1},\lambda_{2}}$ is a translate of $\lambda_{2}(K\times\{0\})$ and can be covered by *m* translates of $(\lambda_{2}\gamma)C=(\lambda_{1}\gamma)C$.

Now we consider the case when $\lambda:=\lambda_{1}\in(0,1)$ and $\lambda_{2}=1$. There exists a set of *m* points $\{{\bar{u}_{i}}: {i\in [m]} \}\subset\mathbb{R}^{n}$ such that

$$ K\subseteq\bigcup _{i\in[m]}(\bar{u}_{i}+\gamma K). $$

Let *z* be an arbitrary point in $C_{\lambda,1}$. Then there exist $\bar{y}\in K$ and $\mu\in[\lambda,1]$ such that $z=\mu(\bar {y},0)+(1-\mu)p$. Lemma 2 shows that there exists $i\in[m]$ such that

$$ z\in\lambda(\bar{u}_{i},0)+(\lambda\gamma+1-\lambda)C. $$

It follows that $C_{\lambda,1}$ can be covered by *m* translates of $(\lambda\gamma+1-\lambda)C$.

When $0<\lambda_{1}<\lambda_{2}\leq1$, (4) shows that $C_{\lambda_{1},\lambda_{2}}$ is a translate of $\lambda_{2}(C_{\lambda_{1}/\lambda_{2},1})$, which can be covered by *m* translates of

$$ \lambda_{2}\biggl(\frac{\lambda_{1}}{\lambda_{2}}\gamma+1-\frac{\lambda _{1}}{\lambda_{2}} \biggr)C=(\lambda_{1}\gamma+\lambda_{2}-\lambda_{1})C. $$

 □

### Theorem 4

*Suppose that* $m\in\mathbb{Z}^{+}$ *and* $\gamma=\Gamma_{m}(K)$. *Then*

$$ \Gamma_{m+1}(C)\leq\frac{1}{2-\gamma}. $$

### Proof

Put $\lambda=1/(2-\gamma)$. Then, by (4), $C_{0,\lambda}$ can be covered by a translate of *λC*, and $C_{\lambda,1}$ can be covered by *m* translates of

$$ (\lambda\gamma+1-\lambda)C=\lambda C. $$

Thus $\Gamma_{m+1}(C)\leq\lambda$. □

### Remark 5

When *K* is a planar convex body, we have that $\Gamma_{7}(K)\leq1/2$. Moreover, if *K* is centrally symmetric, then we always have $\Gamma_{7}(K)=1/2$ (see [11]). Therefore, for a convex cone *C* in $\mathbb{R}^{3}$ having a planar convex body as a base, we have $\Gamma_{8}(C)\leq2/3$, an estimation which was already obtained by Chuanming Zong in [9]. It is also mentioned in [11] that $\Gamma_{7}(K)=5/11$ when *K* is a triangle. Therefore, when *T* is a tetrahedron, we have $\Gamma _{8}(T)\leq 11/17<2/3$; see Table 1, where Δ is a triangle and *C* is a cone whose base is Δ. We have a numerical example showing that the estimation $\Gamma_{8}(T)\leq11/17$ is not optimal either.

**Table 1:** Exact values of $\Gamma_{m}(\Delta)$ (cf. [11, p. 333]) and $\Gamma_{m}(T)$ (cf. [9, p. 2559]) and consequences of Theorem 4 for $\Gamma_{m}(C)$ with $C = T$

*m*	3	4	5	6	7	8
$\Gamma_{m}(\Delta)$	$\frac{2}{3}$	$\frac{4}{7}$	$\frac {8}{15}$	$\frac{1}{2}$	$\frac{5}{11}$	$\frac{3}{7}$
$\Gamma_{m}(T)$	1	$\frac{3}{4}$	$\frac{9}{13}$	–	–	–
$\Gamma_{m}(C)$	1	${\leq}\frac{3}{4}$	${\leq}\frac{7}{10}$	${\leq}\frac{15}{22}$	${\leq}\frac{2}{3}$	${\leq}\frac{11}{17}$

## Covering functionals of double cones

Let $K\subseteq\mathbb{R}^{n}$ be a convex body, *m* be a positive integer such that $\Gamma_{m}(K)<1$, and $p,q\in\mathbb{R}^{n+1}\setminus\mathbb {R}^{n}\times\{0\}$ be two points such that $[{p},{q} ]\cap(K\times\{0\})\neq \emptyset$. The set

$$ C=\operatorname{conv}\bigl(K\times\{0\}\cup\{p,q\}\bigr) $$

is called a *double cone*.

### Theorem 6

*Let* *C* *be a double cone defined as above*. *Then*

$$ \Gamma_{m+2}(C)\leq\frac{1}{2-\Gamma_{m}(K)}. $$

### Proof

Put

$$ \gamma=\Gamma_{m}(K)\quad \text{and}\quad \lambda=\frac{1}{2-\Gamma_{m}(K)}. $$

Then $\lambda\in(1/2,1)$. Without loss of generality we may assume that $[{p},{q} ]$ intersects $K\times\{0\}$ in the origin *o* of $\mathbb{R}^{n+1}$. By applying a suitable non-singular affine transformation if necessary, we may assume that $p=e_{n+1}$ and $q=-\alpha e_{n+1}$, where *α* is a positive number. Suppose that $[{\beta_{1}},{\beta_{2}} ]\subseteq [{-\alpha },{1} ]$. Put

$$ C_{\beta_{1}}^{\beta_{2}}= \bigl\{ {z\in C}: { ({z}|{e_{n+1}} )\in [{\beta_{1}},{\beta_{2}} ]} \bigr\} . $$

Then it is clear that

$$ C=C_{1-\lambda}^{1}\cup C_{-\alpha}^{-(1-\lambda)\alpha}\cup C_{-(1-\lambda)\alpha}^{1-\lambda}. $$

First we show that

$$ C_{1-\lambda}^{1}\subseteq\lambda C+(1-\lambda)e_{n+1}. $$

Let *z* be an arbitrary point in $C_{1-\lambda}^{1}$. If $z=p$, then *z* is clearly in $\lambda C+(1-\lambda)p$. In the following we assume that

$$ 1-\lambda\leq ({z}|{e_{n+1}} )< 1. $$

Then there exist $\lambda_{1},\lambda_{2},\lambda_{3}\geq0$, and $x\in K\times\{0\}$ such that

$$ \sum _{i\in[3]}\lambda_{i}=1, 1-\lambda\leq \lambda_{2}-\alpha \lambda_{3}< 1,\quad \text{and}\quad z= \lambda_{1} x+\lambda_{2} p+\lambda_{3}q. $$

It follows that

5 $$\begin{aligned} z&=\lambda_{1} x+(\lambda_{2}-\alpha\lambda_{3})e_{n+1} \\ &=\lambda_{1} x+(\lambda_{2}-\alpha\lambda_{3}+ \lambda -1)e_{n+1}+(1-\lambda)e_{n+1} \\ &=\lambda\biggl(\frac{1-\lambda_{2}+\alpha\lambda_{3}}{\lambda}\cdot\frac {\lambda_{1}}{1-\lambda_{2}+\alpha\lambda_{3}}x+\frac{\lambda_{2}-\alpha \lambda_{3}+\lambda-1}{\lambda}p \biggr)+(1-\lambda)p. \end{aligned}$$

On the one hand, we have

$$ 0\leq\lambda_{2}-\alpha\lambda_{3}+\lambda-1\leq\lambda, $$

which implies that

6 $$ \frac{\lambda_{2}-\alpha\lambda_{3}+\lambda-1}{\lambda}\in [{0},{1} ]. $$

And on the other hand, we have

$$ 0\leq\lambda_{1}=1-\lambda_{2}-\lambda_{3}\leq1- \lambda_{2}+\alpha \lambda_{3}, $$

which shows that (since $o\in K\times\{0\}$)

7 $$ \frac{\lambda_{1}}{1-\lambda_{2}+\alpha\lambda_{3}}x\in K\times\{0\}. $$

From (5), (6), and (7) it follows that $z\in\lambda C+(1-\lambda)p$.

In the second step we show that

$$ C_{-\alpha}^{-(1-\lambda)\alpha}\subseteq\lambda C+(1-\lambda)q. $$

Similarly, we only need to consider the case when $-\alpha< ({z}|{e_{n+1}} )\leq-(1-\lambda)\alpha$. In this case, there exist $\lambda_{1},\lambda_{2},\lambda_{3}\geq0$, and $x\in K\times\{0\}$ such that

$$ \sum _{i\in[3]}\lambda_{i}=1, -\alpha< \lambda_{2}-\alpha \lambda_{3}\leq-\alpha(1-\lambda),\quad \text{and}\quad z=\lambda_{1} x+\lambda _{2} p+\lambda_{3}q. $$

We have

8 $$\begin{aligned} z&=\lambda_{1} x+\bigl(\lambda_{2}+(1-\lambda)\alpha-\alpha \lambda _{3}\bigr)e_{n+1}-(1-\lambda)\alpha e_{n+1} \\ &=\lambda\biggl(\frac{\lambda_{1}\alpha}{\lambda\alpha}x-\frac{-\lambda _{2}-(1-\lambda)\alpha+\alpha\lambda_{3}}{\lambda\alpha}\alpha e_{n+1} \biggr)-(1-\lambda)\alpha e_{n+1} \\ &=\lambda\biggl(\frac{\lambda_{2}+\alpha-\alpha\lambda_{3}}{\lambda\alpha }\cdot\frac{\lambda_{1}\alpha}{\lambda_{2}+\alpha-\lambda_{3}\alpha }x+\frac{-\lambda_{2}-(1-\lambda)\alpha+\alpha\lambda_{3}}{\lambda \alpha}q \biggr) \\ &\quad{}+(1-\lambda)q. \end{aligned}$$

Moreover, the following inequalities hold:

9 $$\begin{aligned}& \lambda_{2}+\alpha-\alpha\lambda_{3}>0, \end{aligned}$$

10 $$\begin{aligned}& 0\leq\alpha\lambda_{1}=\alpha(1-\lambda_{2}- \lambda_{3})\leq\lambda _{2}+\alpha-\alpha \lambda_{3}, \end{aligned}$$

and

11 $$ 0\leq-\lambda_{2}-(1-\lambda)\alpha+\alpha \lambda_{3}\leq\lambda \alpha. $$

Then (8) and equalities (9), (10), and (11) show that $z\in \lambda C+(1-\lambda)q$.

In the following we assume that $\{{\bar{u}_{i}}: {i\in[m]} \}\subseteq\mathbb{R}^{n}$ is a set of points such that

$$ K\subseteq\bigcup _{i\in[m]}(\bar{u}_{i}+\gamma K). $$

Suppose that $({z}|{e_{n+1}} )\in [{0},{1-\lambda } ]$. Then there exist $\lambda_{1},\lambda_{2},\lambda_{3}\geq0$, $x\in K\times\{0\}$ such that

$$ \sum _{i\in[3]}\lambda_{i}=1, 0\leq \lambda_{2}-\alpha\lambda _{3}\leq1-\lambda, z= \lambda_{1}x+\lambda_{2}p+\lambda_{3}q. $$

In this situation we have

12 $$ z=\lambda_{1}x+(\lambda_{2}-\alpha \lambda_{3})e_{n+1}=(1-\lambda _{2}+\alpha \lambda_{3})\cdot\frac{\lambda_{1}}{1-\lambda_{2}+\alpha \lambda_{3}}x+(\lambda_{2}-\alpha \lambda_{3})e_{n+1}. $$

Moreover,

$$ 0\leq\lambda_{1}=1-\lambda_{2}-\lambda_{3}\leq1- \lambda_{2}+\alpha \lambda_{3}\quad \Rightarrow\quad \frac{\lambda_{1}}{1-\lambda_{2}+\alpha\lambda_{3}} \in [{0},{1} ], $$

which shows that

$$ x':=\frac{\lambda_{1}}{1-\lambda_{2}+\alpha\lambda_{3}}x\in K\times\{0\}. $$

Put

$$ \mu=1-\lambda_{2}+\alpha\lambda_{3}. $$

Then $\mu\in [{\lambda},{1} ]$. From Lemma 2 it follows that $z\in\lambda(\bar{u}_{i},0)+\lambda C$ for some $i\in[m]$. Therefore

$$ C_{0}^{1-\lambda}\subseteq\bigcup_{i\in[m]} \bigl(\lambda(\bar {u}_{i},0)+\lambda C\bigr). $$

It remains to consider the case when $({z}|{e_{n+1}} )\in [{-(1-\lambda)\alpha },{0} ]$. Then there exist $\lambda_{1},\lambda_{2},\lambda_{3}\geq0$, $x\in K\times\{0\}$ such that

$$ \sum _{i\in[3]}\lambda_{i}=1, \qquad -(1-\lambda)\alpha\leq \lambda _{2}-\alpha\lambda_{3}\leq0,\qquad z=\lambda_{1}x+ \lambda_{2}p+\lambda_{3}q. $$

We have

$$\begin{aligned} z&=\lambda_{1}x+(\lambda_{2}-\alpha\lambda_{3})e_{n+1} \\ &=\lambda_{1}x-\frac{-\lambda_{2}+\alpha\lambda_{3}}{\alpha}\alpha e_{n+1} \\ &=\frac{\alpha+\lambda_{2}-\alpha\lambda_{3}}{\alpha}\cdot\frac {\alpha\lambda_{1}}{\alpha+\lambda_{2}-\alpha\lambda_{3}}x+\frac {-\lambda_{2}+\alpha\lambda_{3}}{\alpha}q. \end{aligned}$$

Clearly,

$$ 0\leq\alpha\lambda_{1}=\alpha(1-\lambda_{2}- \lambda_{3})\leq\alpha +\lambda_{2}-\alpha \lambda_{3}, $$

which shows that

$$ x':=\frac{\alpha\lambda_{1}}{\alpha+\lambda_{2}-\alpha\lambda_{3}}x\in K\times\{0\}. $$

Moreover, one can easily verify that

$$ \mu=\frac{\alpha+\lambda_{2}-\alpha\lambda_{3}}{\alpha}\in [{\lambda},{1} ]. $$

Again, by Lemma 2, $z\in\lambda(\bar {u}_{i},0)+\lambda C$ for some $i\in[m]$. Therefore

$$ C_{-(1-\lambda)}^{0}\subseteq\bigcup_{i\in[m]} \bigl(\lambda(\bar {u}_{i},0)+\lambda C\bigr). $$

 □

In Table 2, *C* is a double cone whose base is $K_{1}^{2}$ (by $K_{p}^{n}$ we denote the unit ball of the Banach space $l_{p}^{n}$). Compared with exact values of $\Gamma_{m}(K_{1}^{3})$, the estimations of $\Gamma_{m}(C)$ given by Theorem 6 are optimal for $m=6,7,8$.

**Table 2:** Exact values of $\Gamma_{m}(K_{1}^{2})$ (cf. [11, p. 333] ) and $\Gamma_{m}(K_{1}^{3})$ (cf. [9, p. 2559]) and consequences of Theorem 6 for $\Gamma_{m}(C)$ with $C=K_{1}^{3}$

*m*	3	4	5	6	7	8
$\Gamma_{m}(K_{1}^{2})$	1	$\frac{1}{2}$	$\frac{1}{2}$	$\frac {1}{2}$	$\frac{1}{2}$	$\frac{1}{2}$
$\Gamma_{m}(K_{1}^{3})$	1	1	1	$\frac{2}{3}$	$\frac{2}{3}$	$\frac{2}{3}$
$\Gamma_{m}(C)$	1	1	1	$\leq\frac{2}{3}$	$\leq\frac{2}{3}$	$\leq\frac{2}{3}$

## Conclusion

Let *K* be a convex body in $\mathbb{R}^{n}$ and *C* be a compact convex cone in $\mathbb{R}^{n+1}$ having $K\times\{0\}$ as a base. We proved that

$$ \Gamma_{m+1}(C)\leq\frac{1}{2-\Gamma_{m}(K)}. $$

A similar estimation is also provided for double cones. These estimations are optimal for particular pairs of *m* and *K*, are better than existing estimations, but they are not always optimal.

In the authors’ opinion, it is interesting to do the following: provide better estimations of the covering functionals of cones and double cones, characterize convex bodies that are sufficiently close to cones or double cones via their boundary structure, and, more importantly, get precise values of $\Gamma_{2^{n}}(K)$ when *K* is an *n*-simplex or $K_{1}^{n}$ for $n\geq3$.

## References

[1] Boltyanski V., Martini H., Soltan P.S. (1997). Excursions into Combinatorial Geometry.

[2] Martini H., Soltan V. (1999). Combinatorial problems on the illumination of convex bodies. Aequ. Math..

[3] Brass P., Moser W., Pach J. (2005). Research Problems in Discrete Geometry.

[4] Bezdek K. (2010). Classical Topics in Discrete Geometry.

[5] Bezdek, K., Khan, M.A.: The geometry of homothetic covering and illumination. ArXiv e-prints (2016)

[6] Carl B., Stephani I. (1990). Entropy, Compactness and the Approximation of Operators.

[7] Zong C. (2016). Functionals on the spaces of convex bodies. Acta Math. Sin. Engl. Ser..

[8] Gruber P.M. (1993). The space of convex bodies. Handbook of Convex Geometry, Vols. A, B.

[9] Zong C. (2010). A quantitative program for Hadwiger’s covering conjecture. Sci. China Math..

[10] Bezdek K., Khan M.A. (2016). On the covering index of convex bodies. Aequ. Math..

[11] Lassak M. (1987). Covering plane convex bodies with smaller homothetical copies. Intuitive Geometry.

